# The complete mitochondrial genome of *Tenebroides mauritanicus* Linnaeus, 1758 (Coleoptera: Trogossitidae)

**DOI:** 10.1080/23802359.2023.2189494

**Published:** 2023-03-29

**Authors:** Yue Wang, Bowei Zhou, Yiqi Lin, Mingqing Weng, Zhimin Zhang, Yinghua Tong

**Affiliations:** a College of Forestry, Fujian Agriculture and Forestry University, Fuzhou City, China; b Key Laboratory of Integrated Pest Management in Ecological Forests, Fujian Province University, Fujian Agriculture and Forestry University, Fuzhou City, China; c Minhou County Forestry Bureau Forest Pest Control Station, Fuzhou City, China

**Keywords:** *Tenebroides mauritanicus*, mitochondrial genome, phylogenetic analysis

## Abstract

*Tenebroides mauritanicus* Linnaeus, 1758 (Coleoptera: Trogossitidae) is a storage pest that feeds mainly on soybean and corn. In this study, we sequenced the entire mitochondrial genome of *Tenebroides mauritanicus* (GenBank accession number: OM161967). The total length of the mitochondrial genome is 15,696 bp, GC content is 29.65%, and the contents of each base is 38.37% A, 18.35% C, 11.30% G and 31.98% T, respectively. The genome encodes 13 protein-coding genes (PCGs), 22 transfer RNA genes (tRNAs) and 2 ribosomal RNA genes (rRNAs). Phylogenetic analysis showed that *Tenebroides mauritanicus* is clustered with *Byturus ochraceus*. This study provides a piece of valuable genomic information for the population genetics, phylogeny, and molecular taxonomy of *Tenebroides mauritanicus*.

## Introduction

*Tenebroides mauritanicus* Linnaeus, 1758 belongs to Trogossitidae, a small beetle family in the Polyphaga suborder. It originated in the Africa and has great destructive power (Henríquez-Valido et al., 2020), making it an important storage pest in both high temperate and cold zones. The species generally overwinter as adults in wood boards and sawdust but also as larvae (Shen 1998). The larvae are harmful to soybeans and corn, and their peeling and feces can easily contaminate food, causing great harm (Wu 1997). However, this widespread food pest has been neglected and little research has been done on it. In this study, we sequenced the whole mitochondrial genome of *Tenebroides mauritanicus* and established a phylogenetic tree based on the Maximum Likelihood method to understand the phylogenetic relationships of *Tenebroides mauritanicus*. These results will support important genetic information for future studies related to the genetic evolution of *Tenebroides mauritanicus*.

## Materials and methods

**Figure 1.**
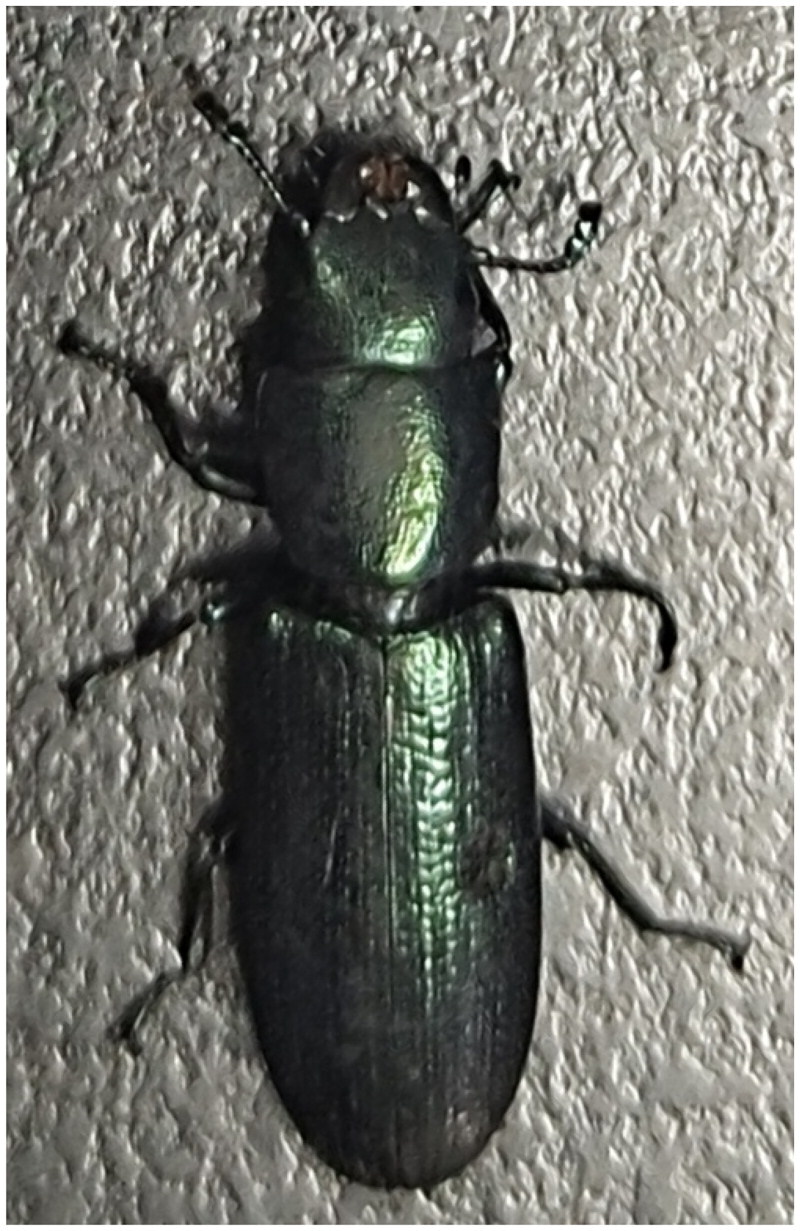
*Tenebroides mauritanicus* Linnaeus, 1758, dorsal view. This is an original image by the authors.

*Tenebroides mauritanicus* was collected using the capturing sex pheromone method in Yangli, Fujian Province, China (118°57′48″E, 26°21′25″N), the specimens were photographed and stored under the specimen number GD-202101 at the Key Laboratory of Integrated Pest Management in Ecological Forests, Fujian Province University, Fujian Agriculture and Forestry University (contact person: Songqing Wu, email: dabinyang@126.com and URL: https://lxy.fafu.edu.cn) (Figure 1). Total genomic DNA was extracted from an adult using the TruSeq DNA Sample Preparation Kit (Vazyme, CHN) and purified with the QIAquick Gel Extraction Kit (Qiagen, GER). DNA purity and concentration were determined with a NanoDrop (Thermo Fisher Scientific, USA). DNA was sequenced with Illumina Hiseq 2500 (Illumina, USA). A total of 713,240 clean reads were obtained from the filtered 59,459,560 raw reads. Clean reads were assembled using metaSPAdes and MitoZ (Nurk et al. 2017). The assembled sequences were then annotated with the MITOs webserver (Matthias et al. 2013). To clarify the accuracy of the assembly, we further mapped our clean reads back to the assemled mitochondrial genome to assess the depth of coverage (Figure S1). Finally, tRNAs genes were predicted with the tRNAscan application (Lowe and Eddy 1997). The whole sequence of the *Tenebroides mauritanicus* mitochondrial genome has been registered in the National Center for Biotechnology Information GenBank and its accession number is OM161967.

## Results

**Figure 2.**
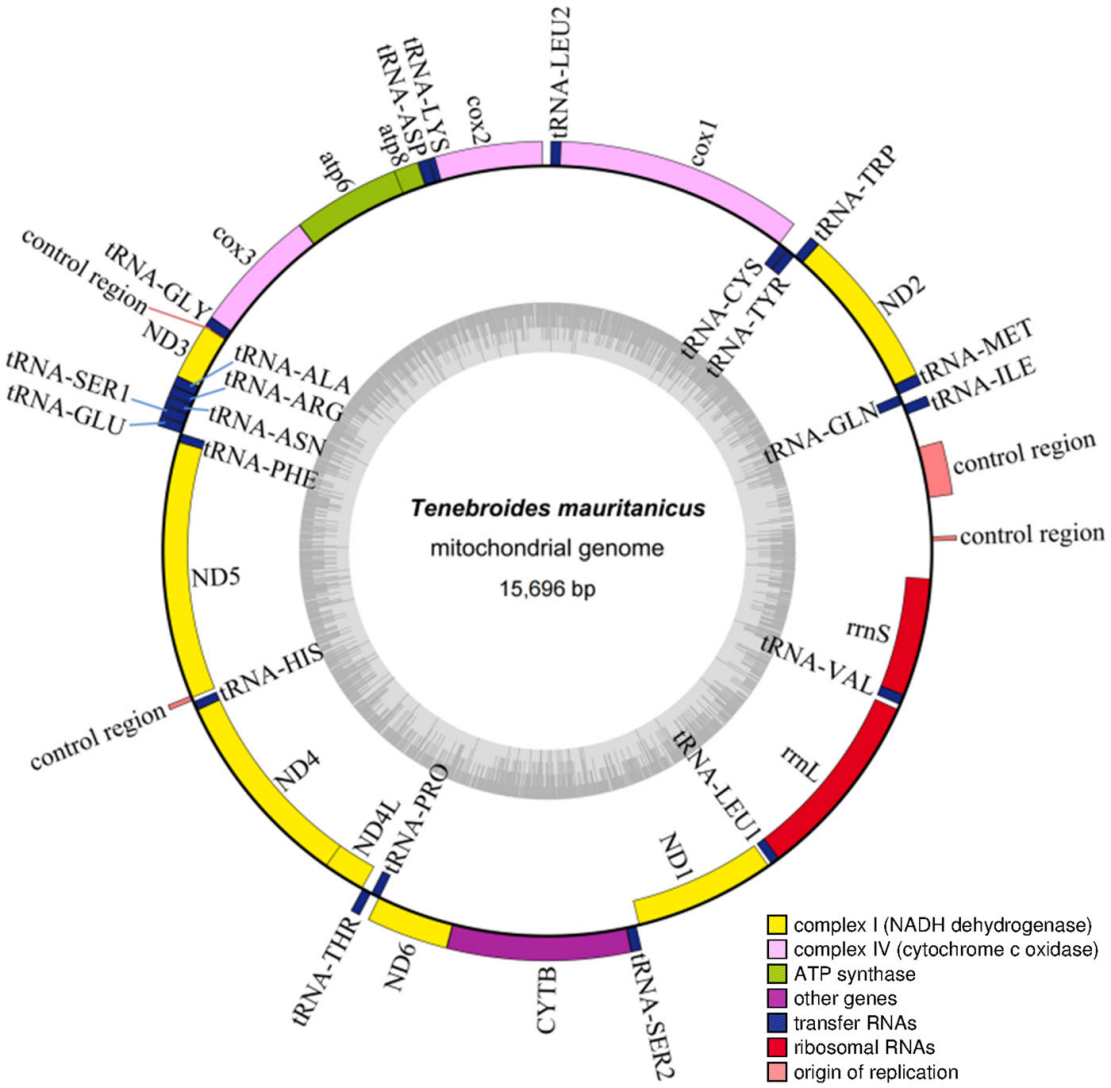
The circular mitogenome map of *Tenebroides mauritanicus.*

The results of the assembly and annotation showed that the whole mitochondrial genome was a 15,696 bp ring structure containing 13 protein-coding genes (PCGs), 22 tRNAs, and 2 rRNAs. The GC content was 29.65%, and A, C, G and T contents were 38.37, 18.35, 11.30, and 31.98%, respectively. The mitochondrial genome map was visualized by using the Organellar Genome DRAW (OGDRAW) (Figure 2). All PCGs (*ATP6, ATP8, COXI, COXII, COXIII, CYTB, ND1, ND2, ND3, ND4, ND4L, ND5*, and *ND6*) comprised a total of 11,049 bp, encoding 3,683 amino acids which six PCGs (*ATP8, ND1, ND2, ND3, ND5, ND6*) beginning with ATT codon, five PCGs (*ATP6, COXIII, CYTB, ND4, ND4L*) with ATG, one PCG (*COXII*) with ATA and one PCG (*COXI*) with TCG. Among them, seven PCGs (*ATP6, ATP8, COXI, COXII, ND2, ND4L, ND6*) stop with a TAA codon and four PCGs (*CYTB, ND1, ND3, ND5*) with TAG. However, the PCGs (*COXIII, ND4*) showed an abnormal stop codon. The rrnS gene was 779 bp, and rrnL gene was 1,272 bp.

**Figure 3.**
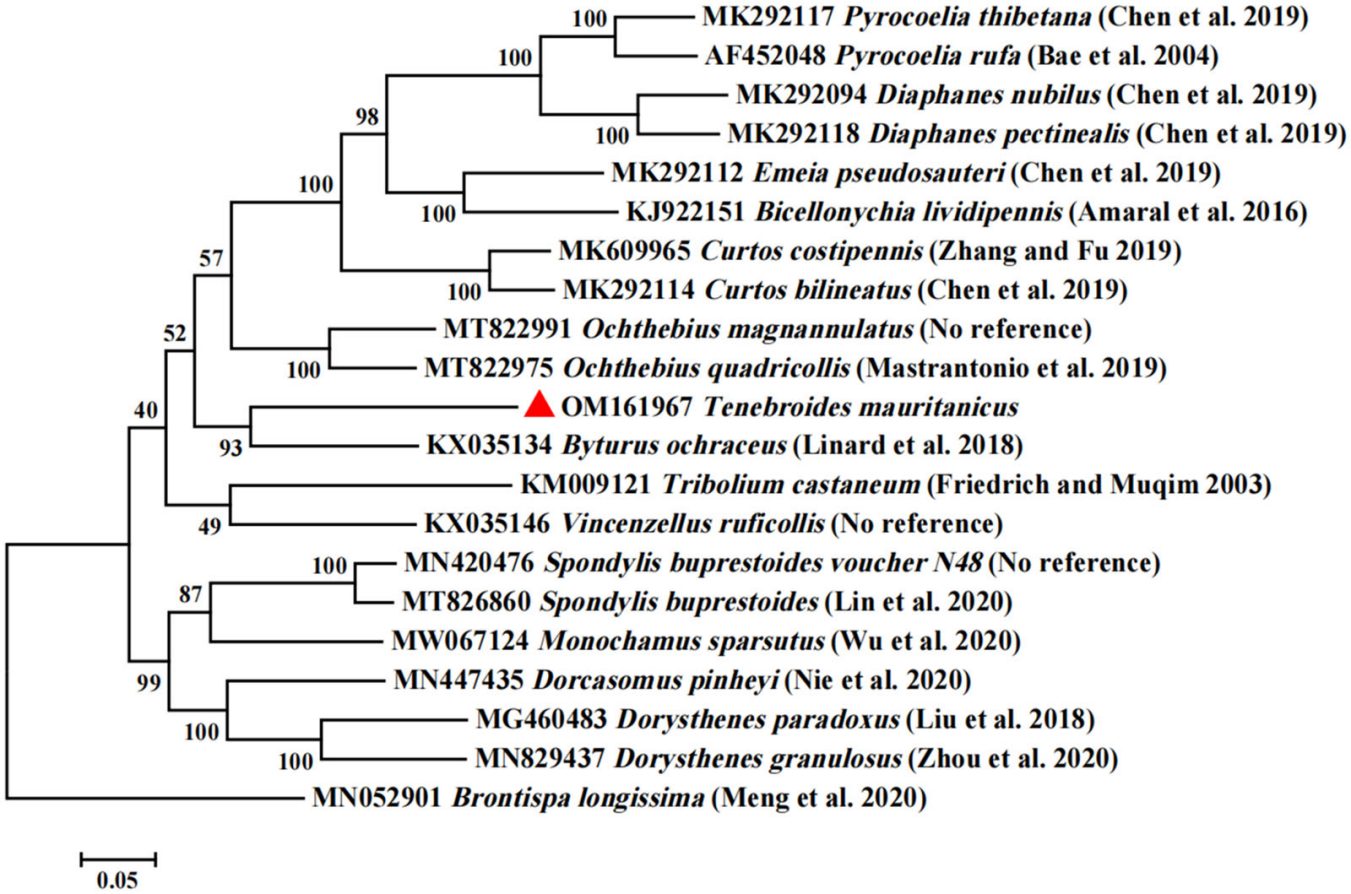
Maximum likelihood tree of *Tenebroides mauritanicus* related to 20 different species of Coleoptera based on the genome sequence. Bootstrap support values are labelled near the branch.

The phylogenetic relationships of *Tenebroides mauritanicus* with related Coleoptera species were confirmed through the creation of an evolutionary tree. The MAFFT 7.0 software was used to compare the sequence of the *Tenebroides mauritanicus* gene to *Brontispa longissima* (GenBank: MN052901) as an out-group (Katoh and Standley 2013). An evolutionary tree was constructed by using MEGA 7 software and using the Maximum Likelihood method and the Tamura-Nei model (Tamura and Nei 1993) with 1000 bootstrap replicates (Kumar et al. 2016). The phylogenetic tree showed that *Tenebroides mauritanicus* was clustered with *Byturus ochraceus* (Figure 3). The complete *Tenebroides mauritanicus* mitochondrial genome has been successfully sequenced and assembled.

## Discussion and conclusions

This study is the first to sequence and annotate the complete mitochondrial genome of *Tenebroides mauritanicus*, and analyze the structural characteristics of the whole mitochondrial genome of *Tenebroides mauritanicus*. The maximum-likelihood tree based on 13PCGs showed that *Tenebroides mauritanicus* was closer to *Byturus ochraceus*. The complete mitochondrial genome published in this study can be used as the basis for further studies on the population genetics, phylogenetic analysis and species identification of *Tenebroides mauritanicus* and related species.

Coleoptera, which has the largest population and contains the most species of insects, has long been the most concerned object of species identification, classification and phylogenetic research. As most of the stored grain pests of Coleoptera are economic pests, special attention is paid to their control (Jiang 2017). Therefore, by exploring the phylogeny, we can explore its genetic evolution from the essence of life, and provide information to develop new strategies for prevention and control of this species.

## Supplementary Material

Supplemental Material

## Data Availability

The genome sequence data that support the findings of this study are openly available in GenBank of NCBI at http://www.ncbi.nlm.nih.gov under the accessment No. OM161967. The associated BioProject, Bio-Sample and SRA number were PRJNA796817, SAMN24922397, and SRR17601604, respectively.
