# Supplementary material for: The complete mitochondrial genome of Tenebroides mauritanicus Linnaeus, 1758 (Coleoptera: Trogossitidae)
Source: Mitochondrial DNA B Resour. 2023 Mar 29;8(3):447–50. doi: 10.1080/23802359.2023.2189494 (PMC10062213; doi:10.1080/23802359.2023.2189494)
Supplement: Supplemental Material [file TMDN_A_2189494_SM3262.docx]

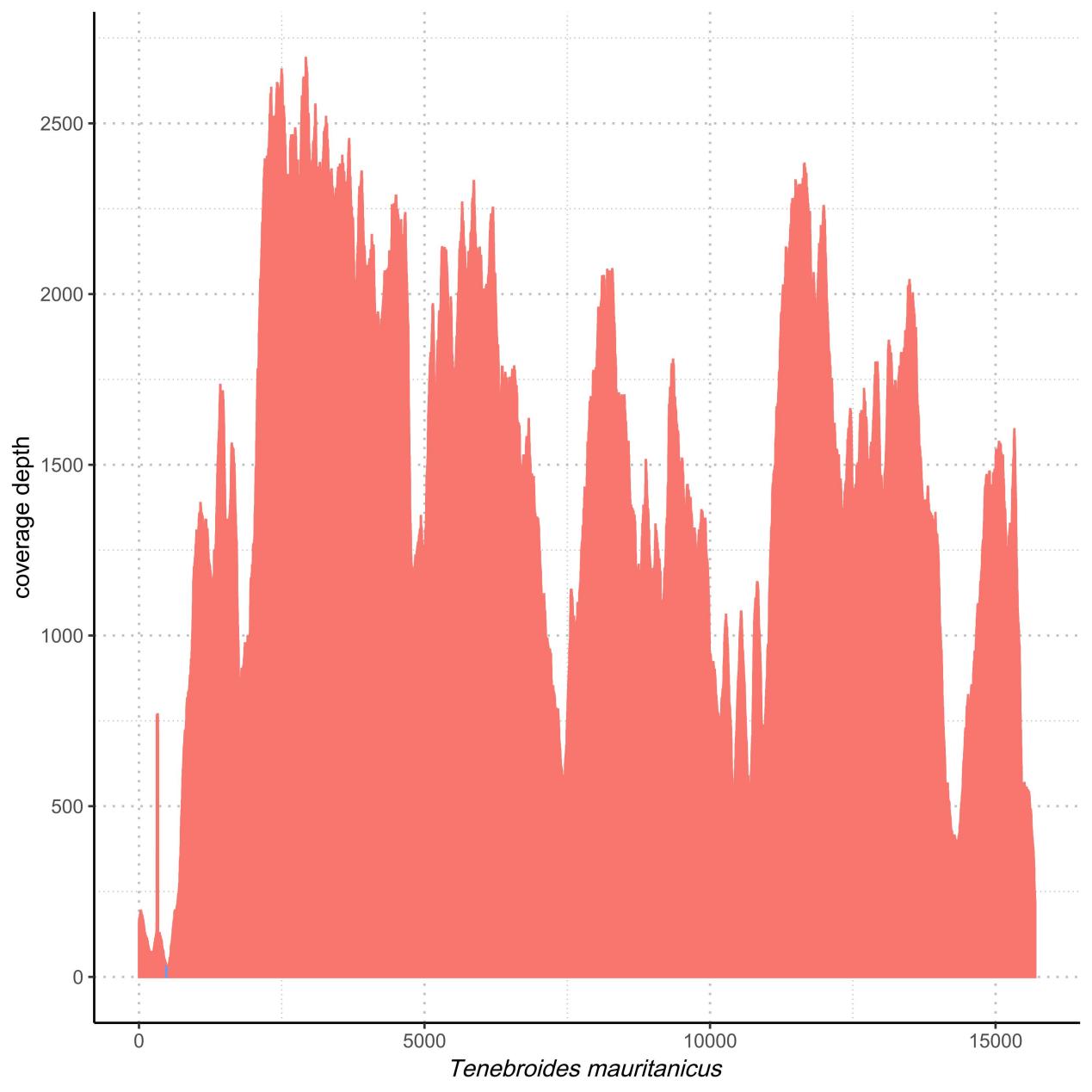


Figure S1. The coverage depth for the complete mitochondrial genome of *Tenebroides mauritanicus.* Blue indicates coverage depth < 30X.
